# Pan-cancer analysis of LRRC59 with a focus on prognostic and immunological roles in hepatocellular carcinoma

**DOI:** 10.18632/aging.205810

**Published:** 2024-05-10

**Authors:** Boyu Pan, Jun Cheng, Wei Tan, Xin Wu, Qizhi Fan, Lei Fan, Minghui Jiang, Rong Yu, Xiaoyun Cheng, Youwen Deng

**Affiliations:** 1 Department of Orthopaedics, The Third Hospital of Changsha, Changsha 410015, Hunan, China; 2 Department of Spine Surgery, The Third Xiangya Hospital, Central South University, Changsha 410013, Hunan, China; 3 Department of Pulmonary and Critical Care Medicine, The Third Xiangya Hospital of Central South University, Changsha 410013, Hunan, China

**Keywords:** LRRC59, tumor microenvironment, immunotherapy, pan-cancer, hepatocellular carcinoma

## Abstract

Background: LRRC59 is a leucine-rich repeats-containing protein located in the endoplasmic reticulum (ER), it serves as a prognostic marker in several cancers. However, there has been no systematic analysis of its role in the tumor immune microenvironment, nor its predictive value of prognosis and immunotherapy response in different cancers.

Methods: A comprehensive pan-cancer analysis of LRRC59 was conducted from various databases to elucidate the associations between its expression and the prognosis of cancer, genetic alterations, tumor metabolism, and tumor immunity. Additionally, further functional assays were performed in hepatocellular carcinoma (HCC) to study its biological role in regulating cell proliferation, migration, apoptosis, cell cycle arrest, and sensitivity to immunotherapy.

Results: The pan-cancer analysis reveals a significant upregulation of LRRC59 in pan-cancer, and its overexpression is correlated with unfavorable prognosis in cancer patients. LRRC59 is negatively correlated with immune cell infiltration, tumor purity estimation, and immune checkpoint genes. Finally, the validation in HCC demonstrates LRRC59 is significantly overexpressed in cancer tissue and cell lines, and its knockdown inhibits cell proliferation and migration, promotes cell apoptosis, induces cell cycle arrest, and enhances the sensitivity to immunotherapy in HCC cells.

Conclusions: LRRC59 emerges as a novel potential prognostic biomarker across malignancies, offering promise for anti-cancer drugs and immunotherapy.

## INTRODUCTION

Cancer represents a foremost public health concern and stands as one of the leading causes of human mortality [1]. Despite substantial advances in medical science, the treatment of cancer still requires improvement, particularly for patients who experience post-operative recurrence or cancer metastasis [2]. Immunotherapy is an emerging cancer treatment approach with huge potential applications, but it still needs to overcome the accompanying side effects [3]. Previous studies have indicated that tumor mutational burden (TMB), immune cell infiltration proportions, and PD-1/PD-L1 expression are well known predictors of cancer immunotherapy response [4]. Nonetheless, these molecular markers have certain limitations in predicting immunotherapy responses in cancer patients [5]. Therefore, there is an urgent need to explore novel biomarkers for assessing responses to these immunotherapeutic approaches, this exploration can aid in the development of more effective immunotherapies, ultimately improving clinical outcomes for cancer patients.

Leucine Rich Repeat Containing 59 (LRRC59) is a protein rich in leucine repeats located in the endoplasmic reticulum and mitochondrial nucleus [6]. It is closely associated with protein misfolding, ER stress, and protein ubiquitination [7]. LRRC59 acts as an intracellular companion protein to FGF1, it interacts with the receptor of FGF1 through its cytoplasmic domain, facilitating the nuclear entry of FGF1 and subsequently inhibiting apoptosis [8, 9]. Similarly, LRRC59 interacts with CIP2A and mediates its nuclear translocation, which promotes deregulation of the cell cycle and increases cancerous phenotypes in prostate cancer [10]. LRRC59 has also been shown in some studies to be significantly upregulated in several cancer tissues and to be related to a poor prognosis [7, 11–15]. Nevertheless, the potential mechanism underlying its antitumor function related to tumor immunity and metabolism remain unclear.

In this study, we conducted a comprehensive analysis of LRRC59 in pan-cancer, detecting its expression and the associations with prognosis, TMB, tumor metabolism, and chemotherapy sensitivity. Additionally, we studied the connection between LRRC59 and immune infiltration, immune-related antigens, and immune checkpoint genes. Further, we validated the biological functions of LRRC59 in hepatocellular carcinoma (HCC). Finally, we established a predictive model based on LRRC59-related genes in HCC, and its accuracy and reliability were validated through external datasets. Our findings assess the role of LRRC59 in tumor immunity, providing valuable insights for its potential as a novel target in tumor immunotherapy.

## MATERIALS AND METHODS

### Data acquisition and differential analysis

The expression of LRRC59 in human cancers was comprehensively analyzed using the TIMER2 (http://timer.comp-genomics.org), GEPIA2 (http://gepia2.cancer-pku.cn), and UALCAN (https://ualcan.path.uab.edu) databases. Additionally, the expression of LRRC59 in normal human tissues were explored using the Harmonizome3 database (https://maayanlab.cloud/Harmonizome). The pan-cancer dataset from the UCSC Xena database (https://xena.ucsc.edu/) and the TCGA-LIHC dataset from the GDC database (https://portal.gdc.cancer.gov/), the LIRI-JP dataset from the ICGC database (https://dcc.icgc.org/), as well as immunohistochemistry data for LRRC59 in HCC patients from the HPA database (https://www.proteinatlas.org/) were acquired and analyzed in this study.

### Prognostic analysis of LRRC59 in pan-cancer

We employed the GEPIA2 database to analyze the correlation between LRRC59 expression and pan-cancer prognosis, including Overall Survival (OS) and Disease-Free Survival (DFS). Additionally, we utilized the Sangerbox (http://sangerbox.com/) to investigate the association of LRRC59 expression with Disease-Specific Survival (DSS) and Progression-Free Survival (PFS) in pan-cancer. The median expression of LRRC59 was used as the grouping cutoff.

### Drugs sensitivity analysis

The CellMiner database (https://discover.nci.nih.gov/cellminer/home.do) was used to obtain RNA expression data for cancer cell lines as well as drug sensitivity data. Clinically tested and FDA-approved drug data were selected for analysis. The correlation analysis was performed using the Spearman method. Missing values in the drug data were imputed using the “impute” package.

### Genetic alterations analysis in pan-cancer

The cBioPortal database (https://www.cbioportal.org/) were utilized to analyze the mutation frequency and genetic alterations of LRRC59 in various cancers. The TIMER2 database was employed to further confirm mutation frequency alterations. The Sangerbox was used for correlation analysis between LRRC59 expression and Human TMB, Mutant-Allele Tumor Heterogeneity (MATH), and Microsatellite Instability (MSI) in pan-cancer. Somatic mutation data related to TCGA-LIHC and ICGC-LIRI datasets were obtained from GDC and ICGC databases, and mutation analysis was conducted using the “maftools” package [16].

### Enrichment analysis

LRRC59 interacting genes were obtained from the STRING database (https://cn.string-db.org/), Gene Ontology (GO) and Kyoto Encyclopedia of Genes and Genomes (KEGG) enrichment analyses were conducted using Metascape database (https://metascape.org). The Hallmark gene sets were acquired from MSigDB database (https://www.gsea-msigdb.org/), and the score of each Hallmark pathway in each in the HCC sample were calculated based on the “GSVA” package [17].

### Clustering and differential analysis

Based on the most significantly enriched Gene Ontology Biological Processes (GO-BP) pathways composed of LRRC59 and its interacting genes obtained from the MSigDB database, clustering analysis were performed on the HCC dataset using the “ConsensusClusterPlus” package [18]. Differential analysis between various clusters was conducted using the “limma” package [19], where genes with logFoldChange>1 and adjusted p-value<0.05 were considered to be statistically different. Next GSEA enrichment analysis was performed using the “clusterProfiler” package [20].

### Construction of HCC prediction model

Based on the clustered genes mentioned above, univariate Cox regression analysis on the TCGA-LIHC dataset was first performed (with survival time greater than 0). Next, three machine learning algorithms (LASSO, randomForest, Xgboost) were employed for further gene selection. Finally, multivariate Cox regression analysis was used to determine the final modeling genes. The accuracy of the model was validated in the ICGC-LIRI dataset.

### Immune infiltration estimation and prediction of immunotherapy outcomes

The correlation between LRRC59 expression and immune cell infiltration, tumor purity estimation, and immune checkpoint expression in various cancers was analyzed using the TIMER2 database and Sangerbox. 11 immune therapy cohorts were obtained from the GEO database (https://www.ncbi.nlm.nih.gov/geo/) and analyzed for the differences in LRRC59 expression among different response groups, as well as its predictive accuracy. The “IOBR” package was utilized to analyze the differences in immune cell infiltration and tumor purity estimation among different subtypes in the HCC dataset [21]. Additionally, The TIDE online website (http://tide.dfci.harvard.edu/) was used to evaluate the response of different HCC subtypes to immunotherapy, and the “Submap” algorithm was used to assess the response of each subtype to TACE treatment (GSE104580) and Sorafenib treatment (GSE109211) [22].

### Cell culture and transfection

The cell lines (LO2, HuH-7, Hep-G2, JHH-5, SNU-387, and SUN-449) were obtained from Xiangya Medical College Cell Bank (Changsha, China), and were cultured according to previously reported methods [23]. shRNAs were acquired from Genechem (Shanghai, China), and cell transfection was carried out following recommended procedures. The shRNA sequences are as follows:

shLRRC59#1, 5’-CCTGGATCTGTCTGTCTTGTAATAA-3’;

shLRRC59#2, 5’-GCAGTTAAAGCAGTGCAAA-3’;

shNC, 5’-AATACGGCGATGTGTCAGG-3’.

### Real-time quantitative PCR

The PrimeScript RT Reagent Kit (TaKaRa, Shiga, Japan) was employed for cDNA synthesis. SYBR Premix ExTaq (TaKaRa, Japan) was used for qPCR. The mRNA primers were as follows:

LRRC59, Forward 5’-TGACTACTCTACCGTCGGATTT-3′,

Reverse 5’-TTCAGGTCCAACCACTTCAGG-3′;

Actin, Forward 5’-ACGCCAACACAGTGCTGTCTG-3′,

Reverse 5’-GGCCGGACTCGTCATACTCC-3′.

### Western blot, CCK-8, ELIS, clone formation assay, wound healing, and transwell assays

The procedures for the above experiments have been described in detail in a previous study [23]. The following antibodies were used: LRRC59 (1:1000; Proteintech, Rosemont, IL, USA), Actin (1:5000, Proteintech), BCL-2 (1:1000; Proteintech), BAX (1:1000; Proteintech).

### Cell cycle analysis and apoptosis detection

Cell cycle analysis was conducted using the Cell Cycle Analysis Kit (BD Biosciences, Shanghai, China) and measured according to its protocol. In brief, 1×10^6^ stable transfected cells were fixed in 70% ethanol for 24 hours, then washed twice with PBS, and stained with propidium iodide (PI) in the dark for 30 minutes, finally subjected to flow cytometric detection. To detect apoptosis, the Annexin V-FITC Apoptosis Detection Kit (BD Pharmingen, La Jolla, CA, USA) was used. Briefly, 1×10^6^ stable transfected cells were suspended in 200 μl binding buffer containing 5 μl Annexin V-FITC and 10 μl PI stain, incubated the cells for 30 minutes in the dark, and finally flow cytometric detection was performed.

### ProcartaPlex multiple immunoassays and T cell-mediated tumor cell-killing assays

Cell culture supernatants were collected and centrifuged for detecting the multiple cytokines and chemokines using the Human Cytokine and Chemokine 34-Plex ProcartaPlex Panel 1A kit and Luminex detection platform (Thermo Fisher Scientific, Waltham, MA, USA) according to manufacturer’s instruction. T cell was extracted from the peripheral blood of healthy donors who gave written informed consent, Dynabeads™ Untouched™ Human CD8 T Cells Kit (Thermo Fisher Scientific, Waltham, MA, USA) was used to extract the T cells, and the detailed steps are performed based on the recommended protocol. 1×10^6^ HCC cells were seeded in 12-well plate overnight, then co-culture with T cells for 48h, wash the cell with PBS twice and then stain the left cells with crystal violet, finally a microplate reader was used to detect OD values at 570 nm.

### Statistical analysis

All data analyses in this study were conducted using R (version 4.2.1) and GraphPad Prism 9. The t test was used for normal distribution, the Wilcoxon rank sum test was used for non-normal distribution, and the Kruskal-Wallis was used for comparison between two or more groups of data. Univariate Cox proportional hazard regression was utilized to examine the relevance between PCD index and overall survival, while multivariate Cox regression was employed to evaluate the independent prognostic significance of PCD index compared to other clinical parameters. A two-way ANOVA test was used to determine the effect of LRRC59 and T cell on cell survival. p<0.05 indicates a statistical difference.

### Availability of data and material

The datasets generated and/or analyzed during the current study are available from the corresponding author upon reasonable request.

## RESULTS

### LRRC59 is overexpressed in pan-cancers

**Figure 1:**
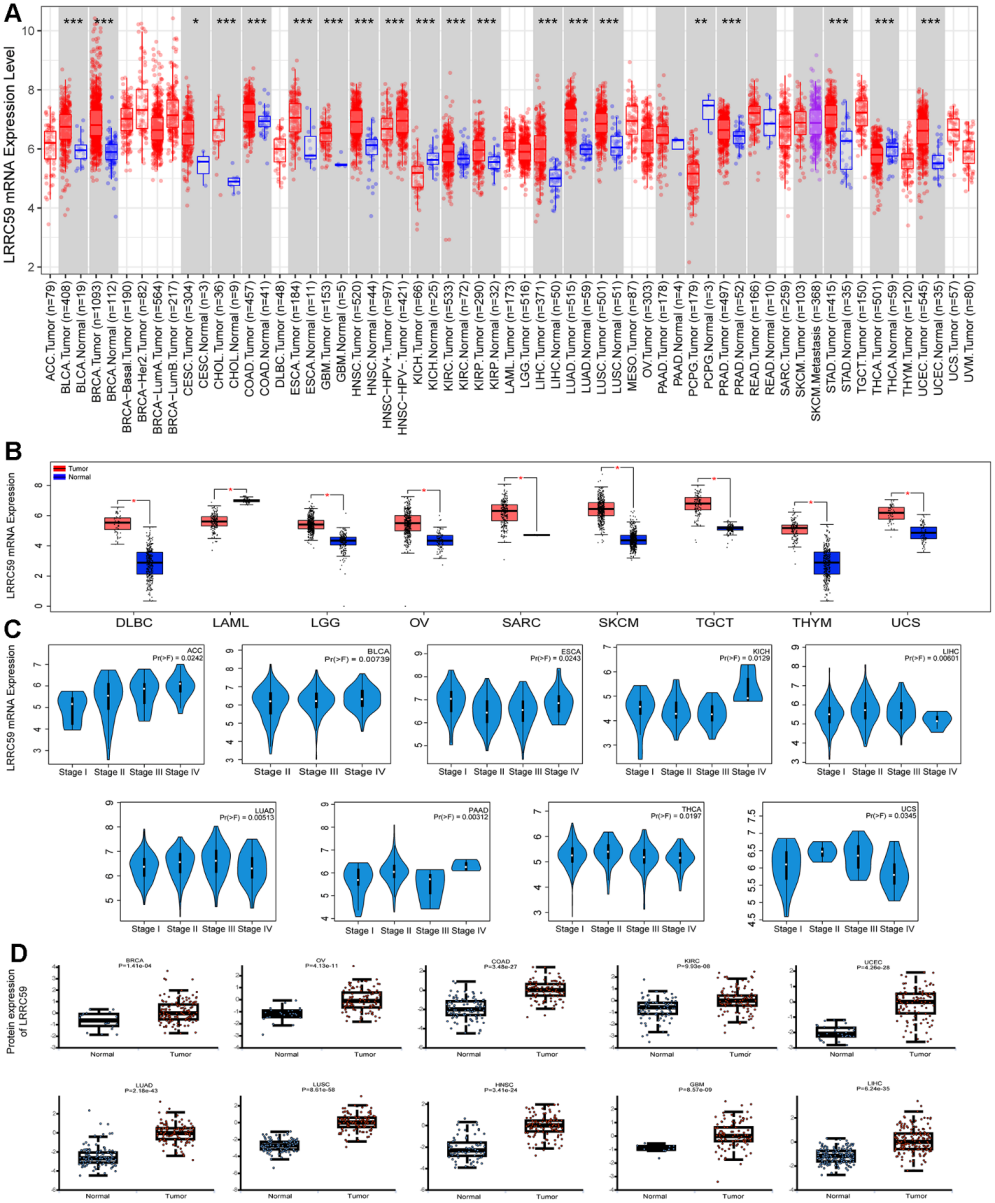
**LRRC59 is overexpressed in cancer tissues.** (**A**, **B**) The expression of LRRC59 in pan-cancer was analyzed in the TIMER2 and GEPIA2 databases. (**C**) The expression of LRRC59 in different pathological stages of pan-cancer. (**D**) Protein expression levels of LRRC59 in pan-cancer.

Analysis from the Harmonizome3 database indicated that LRRC59 can be detected in human normal tissues (Supplementary Figure 1). Then analysis from the TIMER2 database revealed that LRRC59 was significantly upregulated in multiple cancers compared to the adjacent normal tissues, including UCEC, STAD, PRAD, LUAD, LUSC, LIHC, KIRC, KIRP, ESCA, GBM, HNSC, CESC, CHOL, COAD, BLCA, BRCA, only in KICH, PCPG and THCA, LRRC59 was downregulated (Figure 1A). As some cancers lacked corresponding adjacent normal tissue data in the TCGA dataset, we analyzed LRRC59’s expression using the GEPIA2 database for a comprehensive exploration. The results showed that LRRC59 was significantly upregulated in DLBC, LGG, OV, SARC, SKCM, TGCT, THYM, UCS, only LAML showed higher expression in adjacent normal tissues compared to the corresponding cancer tissues (Figure 1B). Pathological staging data about the cancers showed differential expression of LRRC59 in ACC, BLCA, ESCA, KICH, LIHC, LUAD, PAAD, THCA and UCS (Figure 1C). Analysis from the UALCAN database demonstrated that LRRC59 protein levels was elevated in BRCA, OV, COAD, KIRC, UCEC, LUAD, LUSC, HNSC, GBM and LIHC than in normal tissues (Figure 1D). The full names of all cancer abbreviations are listed in Table 1.

**Table 1:** The abbreviations of different cancer types.

**Cancer type**	**Abbreviation**
Adrenocortical Cancer	ACC
Bladder Cancer	BLCA
Breast Cancer	BRCA
Cervical Cancer	CESC
Bile Duct Cancer	CHOL
Colon Cancer	COAD
Large B-cell Lymphoma	DLBC
Esophageal Cancer	ESCA
Glioblastoma	GBM
Head and Neck Cancer	HNSC
Kidney Chromophobe	KICH
Kidney Clear Cell Carcinoma	KIRC
Kidney Papillary Cell Carcinoma	KIRP
Acute Myeloid Leukemia	LAML
Lower Grade Glioma	LGG
Liver Cancer	LIHC
Lung Adenocarcinoma	LUAD
Lung Squamous Cell Carcinoma	LUSC
Mesothelioma	MESO
Ovarian Cancer	OV
Pancreatic Cancer	PAAD
Pheochromocytoma and Paraganglioma	PCPG
Prostate Cancer	PRAD
Rectal Cancer	READ
Sarcoma	SARC
Melanoma	SKCM
Stomach Cancer	STAD
Testicular Cancer	TGCT
Thyroid Cancer	THCA
Thymoma	THYM
Endometrioid Cancer	UCEC
Uterine Carcinosarcoma	UCS
Ocular melanomas	UVM

We analyzed the discriminative ability of LRRC59 for tumor samples using the TCGA pan-cancer dataset provided by the UCSC Xena database as well, the results showed that the diagnostic AUC value of LRRC59 exceeded 0.5 in 19 cancers and exceeded 0.9 in 4 cancers (Supplementary Figure 2). In summary, LRRC59 is significantly upregulated in most cancers and demonstrates high diagnostic efficacy.

### Elevated LRRC59 expression correlates with poor prognosis and chemoresistance in multiple cancers

**Figure 2:**
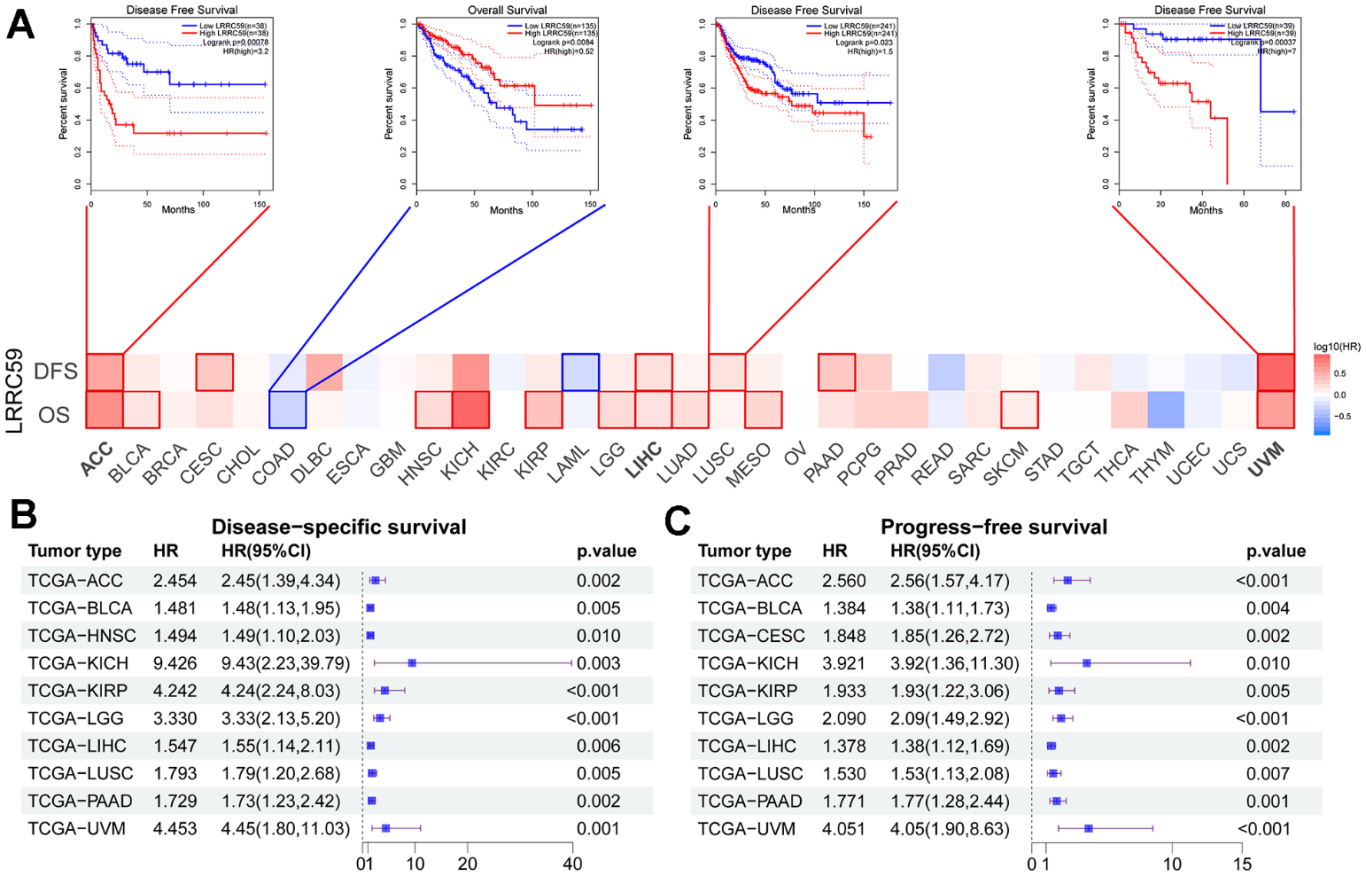
**High expression of LRRC59 indicates a poor prognosis for pan-cancer.** (**A**–**C**) Prognostic analysis of LRRC59 in pan-cancer DFS, OS, DSS and PFS.

Analysis from the GEPIA2 database revealed that high expression of LRRC59 was associated with shorter OS in 11 cancer types, including ACC, BLCA, HNSC, KICH, KIRP, LGG, LIHC, LUAD, MESO, SKCM, and UVM. Only in COAD, patients with high LRRC59 expression had longer OS (Figure 2A). Moreover, patients with high LRRC59 expression had shorter DFS in ACC, CESC, LIHC, LUSC, PAAD and UVM, but longer DFS only in LAML (Figure 2A). Similar findings were obtained from the analysis using Sangerbox, indicating that high LRRC59 expression was closely related to shorter DSS and PFS in various cancers (Figure 2B, 2C). In summary, high LRRC59 expression serves as an effective prognostic marker for poor outcomes in multiple cancers. The analysis of drug sensitivity demonstrated that among the top 16 drugs most correlated with LRRC59 expression, 14 drugs showed a positive correlation with IC50 values, indicating that these drugs may be more ineffective in patients with high LRRC59 expression, only Vinorelbine and Kahalide F exhibited a negative correlation with IC50 values (Supplementary Figure 3).

### Mutation landscape of LRRC59 in various cancers

**Figure 3:**
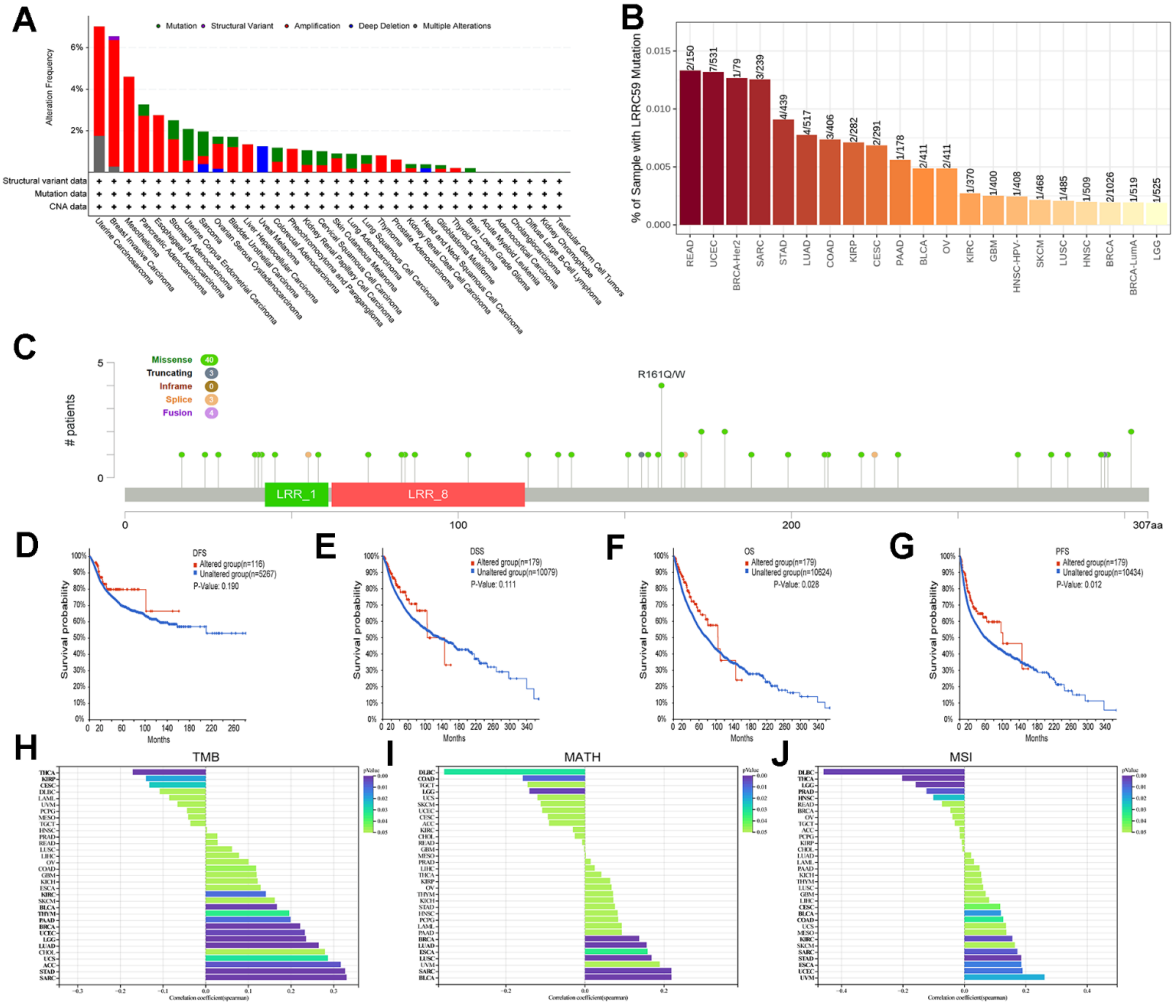
**Mutational landscape of LRRC59 in pan-cancer.** (**A**, **B**) Genetic alterations of LRRC59 in pan-cancer were analyzed in the cBioPortal and TIMER2 databases. (**C**) Visualization of LRRC59 mutation sites in pan-cancer. (**D**–**G**) Analysis of the LRRC59 gene alterations on the prognosis of DFS, DSS, OS and PFS in pan-cancer. (**H**–**J**) Correlation analysis of LRRC59 with TMB, MATH and MSI in pan-cancer.

Analysis of the TCGA pan-cancer dataset in the cBioPortal database revealed genetic alterations of LRRC59 in multiple cancers. The highest alteration frequency was observed in UCS, followed by BRCA and MESO, and the most common type of genetic alteration was “amplification” (Figure 3A). Consistent with these findings, TIMER2 database also showed genetic mutations of LRRC59 in various cancers, with READ exhibiting the highest mutation rate, followed by UCEC and BRCA (Figure 3B). A lollipop chart was used to visualize all mutation sites of LRRC59, with missense mutations being the most common mutation sites (Figure 3C). Survival analysis demonstrated that patients with genetic alterations in LRRC59 had longer DFS and DSS compared to those without genetic alterations (Figure 3D-3E). Additionally, they also exhibited longer OS and PFS (Figure 3F, 3G).

The levels of TMB, MSI, and MATH are considered closely related to the therapeutic response of cancer patients [24]. Thus, the correlation between LRRC59 expression and these factors were analyzed using Sangerbox. The results showed that LRRC59 expression was positively correlated with TMB in SARC, STAD, ACC, UCS, LUAD, LGG, UCEC, BRCA, PAAD, THYM, BLCA, and KIRC, while negatively correlated with TMB in THCA, KIRP, and CESC (Figure 3H). it was also positively correlated with MATH in BLCA, SARC, LUSC, ESCA, LUAD, and BRCA, but negatively correlated with DLBC (Figure 3I). Furthermore, LRRC59 expression was positively correlated with MSI in UVM, UCEC, ESCA, STAD, SARC, KIRC, COAD, BLCA, and CESC, while a negative correlation with DLBC (Figure 3J).

### LRRC59 is associated with pan-cancer immune infiltration and immunotherapy

**Figure 4:**
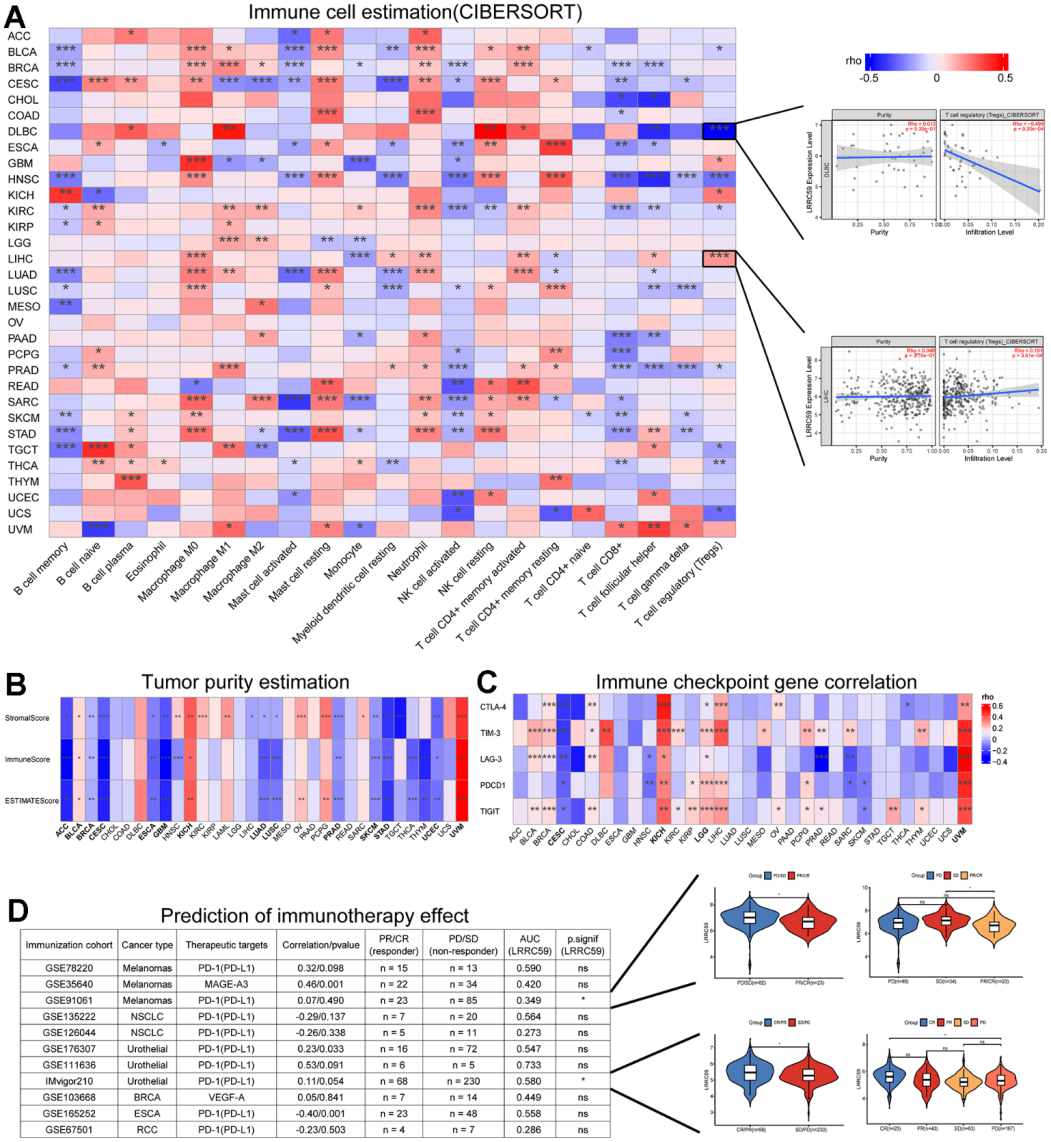
**LRRC59 is associated with immune infiltration and immunotherapy.** (**A**) Correlation analysis of LRRC59 with immune cells in pan-cancer. (**B**) Correlation analysis of LRRC59 with tumor purity estimation in pan-cancer. (**C**) Correlation analysis of LRRC59 with immune checkpoint genes in pan-cancer. (**D**) Correlation analysis of LRRC59 with immunotherapy cohorts in pan-cancer.

The correlation between LRRC59 expression and immune cell infiltration in pan-cancer were analyzed using the CIBERSORT algorithm provided by the TIMER2 database, the results indicated that LRRC59 expression was positively correlated with neutrophils, resting mast cells, and M0 macrophages in most cancers, while it was negatively correlated with memory B cells, activated mast cells, and activated NK cells (Figure 4A). The tumor microenvironment is complex, and we used Sangerbox to analyze the immune and stromal compositions in pan-cancer. The analysis results showed that LRRC59 expression was negatively correlated with ESTIMATE Score in most cancers, indicating a positive correlation with tumor purity in pan-cancer (Figure 4B).

Targeting immune checkpoints has shown promising results in some cancers [25]. Therefore, the correlation between LRRC59 and common immune checkpoints, including CTLA-4, TIM-3, LAG-3, PDCD1, and TIGIT were analyzed in our project. The results revealed that LRRC59 expression was positively correlated with TIM-3 and TIGIT expression, while negatively correlated with LAG-3 and PDCD1 (Figure 4C). For patients with high expression of LRRC59, immunotherapy targeting TIM-3 and TIGIT may be more effective. We then analyzed the correlation between LRRC59 and corresponding checkpoints in 11 immunotherapy cohorts, as well as the expression differences of LRRC59 among different immunotherapy groups. It showed that the AUC values of LRRC59 in predicting immunotherapy response exceeded 0.5 in 6 immunotherapy cohorts, in the IMvigor210 cohort (BLCA), the AUC value was 0.580, which meant LRRC59 expression was relatively higher in the immunotherapy response group (CR/PR). In the GSE91061 cohort (Melanomas), the AUC value was 0.349, which meant LRRC59 expression was higher in the immunotherapy resistance group (PD/SD) (Figure 4D).

### Poor prognosis in HCC with high LRRC59 expression

20 genes that interact with LRRC59 were obtained from the STRING database (Supplementary Figure 4A). Enrichment analysis showed that the most relevant biological processes (BP) of LRRC59 and its interacting genes were the ER-associated degradation pathway (ERAD) and negative regulation of translation (Supplementary Figure 4B), the most relevant molecular functions (MF) were carbohydrate binding (Supplementary Figure 4C), the most relevant cellular components (CC) were rough ER (Supplementary Figure 4D). The most relevant KEGG pathway was Protein processing in the ER (Supplementary Figure 4E). The above enrichment analysis results indicate that LRRC59 may play an important role in protein synthesis and degradation. Given that the liver contains large number of enzymes responsible for the metabolism, and is an important site for protein synthesis in the human body [26], we speculate that LRRC59 dysfunction may play a crucial role in the progression of HCC.

**Figure 5:**
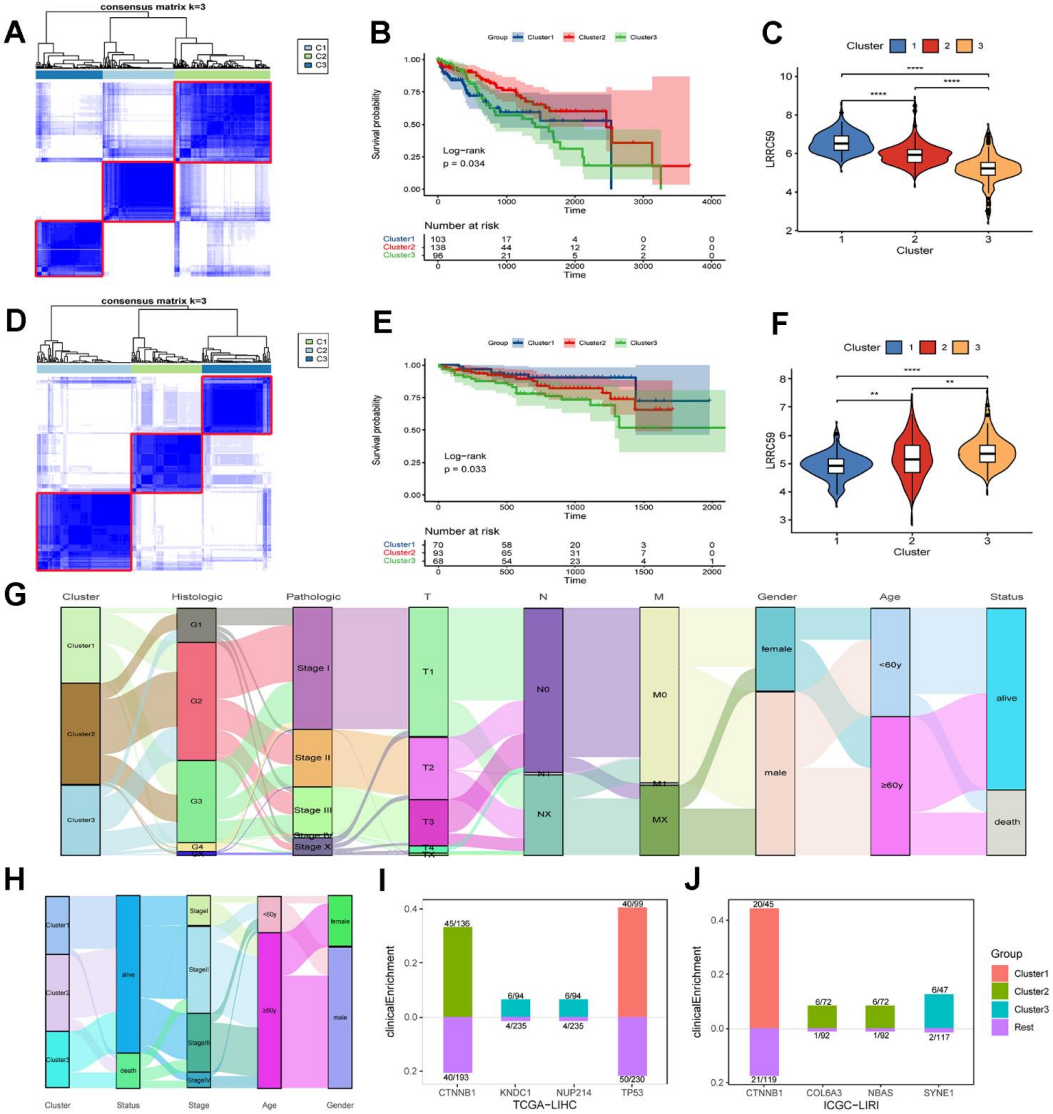
**The prognosis of HCC subtypes with LRRC59 overexpression is poor.** (**A**–**C**) The TCGA-LIHC dataset was clustered and subtyped, and the relationship between LRRC59 expression and prognosis in each subtype was analyzed. (**D**–**F**) The ICGC-LIRI dataset was clustered and subtyped, and the relationship between LRRC59 expression and prognosis in each subtype was analyzed. (**G**, **H**) Visualization of clinical characteristics of each HCC subtype. (**I**, **J**) Clinical enrichment analysis of mutations in each HCC subtypes.

The results from the HPA database showed weak expression of LRRC59 in normal liver tissue, while it was strongly expressed in HCC (Supplementary Figure 5A). In the TCGA-LIHC dataset, paired expression analysis revealed that the expression of LRRC59 in cancer tissues is elevated significantly (Supplementary Figure 5B). Survival analysis and prediction showed that patients with high LRRC59 expression had shorter survival times in liver cancer (Supplementary Figure 5C, 5D). Similar conclusions were drawn from the ICGC-LIRI dataset (Figure 5E–5G). A total of 108 ER-related degradation pathway component genes were obtained from the MSigDB database (Supplementary Table 1), and then screened in the TCGA-LIHC dataset through univariate Cox regression analysis, three machine learning algorithms, and multivariate Cox regression analysis, finally two genes, HM13 and MAN1A1, were obtained (Supplementary Figure 5H–5M). The risk score of each sample was calculated based on the sum of the products of the multivariate Cox regression coefficient and gene expression. The results showed that the prediction model based on the two genes has relatively accurate prediction performance in both the TCGA-LIHC and ICGC-LIRI datasets, their AUC values for survival prediction in 1, 2, and 3 years all exceeded 0.6 (Supplementary Figure 5N–5S).

HCC datasets were clustered based on the ERAD genes, and the differences in LRRC59 expression and clinical characteristics of various HCC subtypes were investigated. In both TCGA-LIHC and ICGC-LIRI datasets, the subtype with high LRRC59 expression had poor prognosis (Figure 5A–5F). The alluvial diagram revealed that patients with cluster1 had the highest proportion of pathological grade G4 in the TCGA-LIHC dataset (Figure 5G), and patients with cluster3 had the highest mortality rate in the ICGC-LIRI dataset, and these individuals were concentrated in advanced stages of cancer (stage II-IV) (Figure 5H). Mutation clinical enrichment analysis was performed on the three subtypes in the TCGA-LIHC dataset, and it showed that the subtype with high LRRC59 expression was most related to TP53 mutations (Figure 5I), and the subtype with high LRRC59 expression was most associated with SYNE1 mutations in the ICGC-LIRI dataset (Figure 5J).

### Cell cycle regulation in high LRRC59-expressing HCC

**Figure 6:**
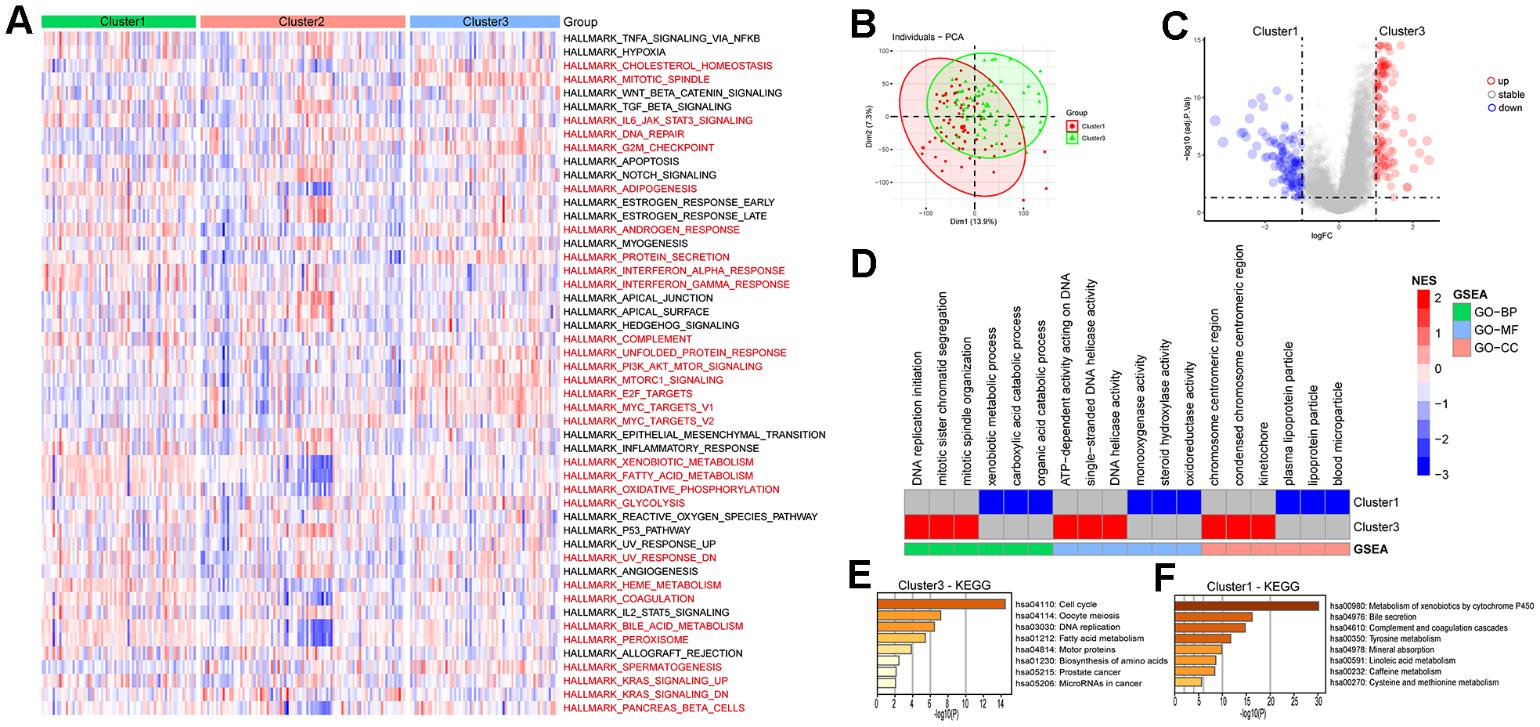
**Enrichment analysis of HCC subtypes.** (**A**) Analysis of Hallmark gene set score differences in the HCC subtypes. (**B**, **C**) Differential genetic analysis of HCC in cluster1 and cluster3. (**D**) Enrichment analysis of differential genes in HCC cluster1 and cluster3. (**E**, **F**) KEGG enrichment analysis of differential genes in HCC cluster1 and cluster3.

The pathway scores for each subtype in the ICGC-LIRI dataset were calculated based on the 50 Hallmark pathway gene sets provided by the MSigDB database, the results showed that there were 30 pathways among each subtype in total (Figure 6A). In the HALLMARK G2M CHECKPOINT pathway, Cluster3 had the highest score, while Cluster1 had the lowest, indicating a positive correlation between LRRC59 and the G2M CHECKPOINT pathway (Supplementary Figure 6A). The subtype with high LRRC59 expression in the ICGC-LIRI dataset was Cluster3, which had the poorest prognosis, while Cluster1 had the lowest LRRC59 expression level and the best prognosis. Therefore, we conducted differential analysis and GSEA enrichment analysis between the two groups. The results showed that Cluster3 was associated with DNA replication processes, while Cluster1 was associated with metabolic pathways such as xenobiotic metabolic process, monooxygenase activity (Figure 6B–6D). Further KEGG enrichment analysis was performed separately for upregulated and downregulated expressed genes, it indicated that Cluster3 was most closely associated with cell cycle pathway (Figure 6E), while Cluster1 was closely associated with Metabolism of xenobiotics by cytochrome P450 (Figure 6F).

### Poor immunotherapeutic efficacy in LRRC59 overexpression HCC

**Figure 7:**
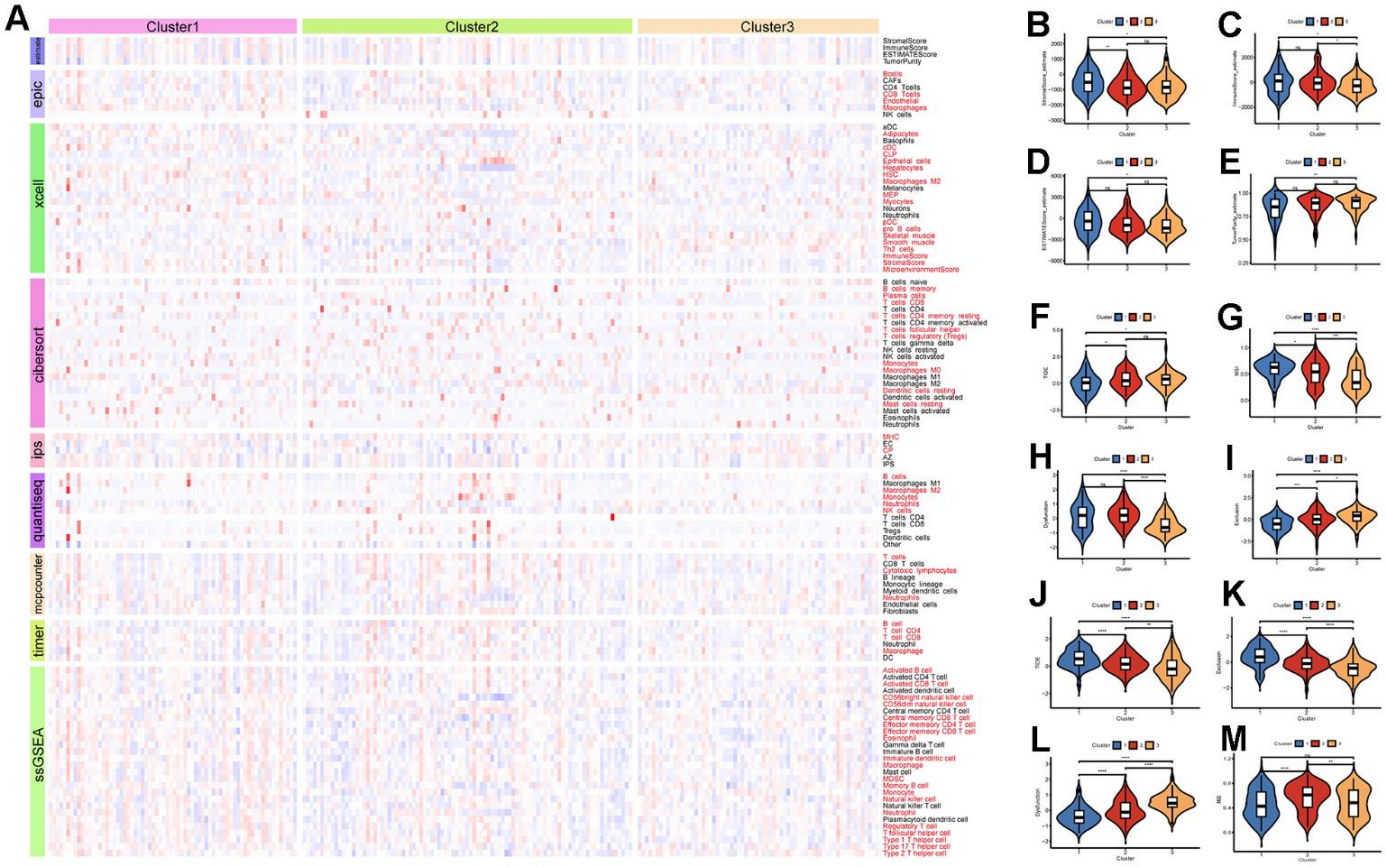
**Immune infiltration and therapeutic analysis of HCC subtypes.** (**A**) Analysis of immune cell infiltration in HCC subtypes. (**B**–**E**) Estimation of tumor purity for HCC subtypes. (**F**–**I**) Analysis of the effect of immunotherapy on HCC subtypes in the ICGC-LIRI dataset. (**J**–**M**) Analysis of the effect of immunotherapy on HCC subtypes in the TCGA-LIHC dataset.

The eight immune cell infiltration algorithms of the “IOBR” package were used to analyze the differences in immune cell infiltration between subtypes in the ICGC-LIRI dataset. The results showed that there are various differences in immune cell infiltration levels among the three subtypes (Figure 7A). Specifically, the CIBERSORT analysis showed that Dendritic resting cells and T regulatory cells (Tregs) had higher infiltration levels in Cluster3, while Monocytes had higher infiltration levels in Cluster1. This is consistent with the previous analysis based on TCGA data about immune cell infiltration estimates in various cancers (Supplementary Figure 6B). Tumor purity analysis demonstrated that Cluster3 had lower stromal scores, immune scores, and ESTIMATE scores, indicating that the tumor purity is higher, which is also consistent with the previous analysis (Figure 7B–7E). According to the data provided by the TIDE online tool, Cluster3 in the ICGC-LIRI dataset had higher TIDE scores and lower MSI scores, indicating that Cluster3 may not respond well to immunotherapy, while Cluster1 may be more sensitive to immunotherapy (Figure 7F–7I). Similarly, the subtype with high LRRC59 expression had the highest TIDE scores and Exclusion scores indicating a poor response to immunotherapy in the TCGA-LIHC dataset (Figure 7J–7M).

Additionally, the “Submap” algorithm was used to predict the efficacy of TACE and Sorafenib treatments for different subtypes of HCC. The results showed that in the ICGC-LIRI dataset, Cluster3 was associated with insensitivity to TACE treatment, while Cluster1 was associated with sensitivity to TACE treatment (Supplementary Figure 7A). Cluster1 was also associated with insensitivity to Sorafenib treatment (Supplementary Figure 7B). Analysis in the TCGA-LIHC dataset revealed that HCC with low LRRC59 expression were associated with sensitivity to TACE treatment (Supplementary Figure 7C), and insensitivity to Sorafenib treatment (Supplementary Figure 7D).

### LRRC59 knockdown inhibits HCC cells proliferation and migration

**Figure 8:**
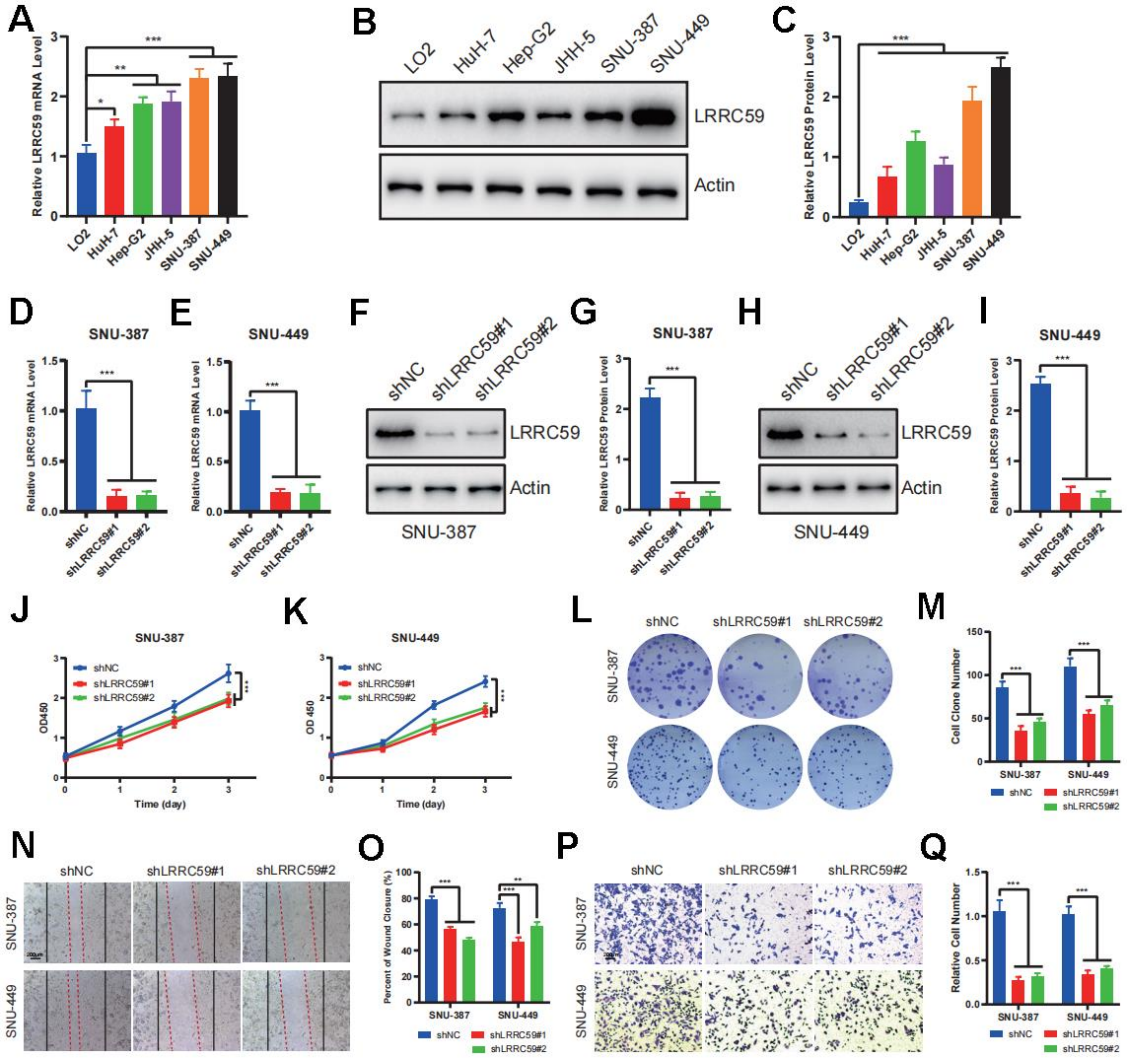
**LRRC59 promotes the proliferation and migration in HCC cells.** (**A**–**C**) The expression of LRRC59 was measured by qRT-PCR and western blot in HCC cells compared to normal liver cell LO2. (**D**, **E**) LRRC59 knockdown was verified by qRT-PCR in SNU-387 and SNU-449. (**F**–**I**) LRRC59 knockdown was verified by western blot in SNU-387 and SNU-449. (**J**, **K**) CCK-8 assays were used to evaluated the proliferation ability of SNU-387 and SNU-449. (**L**, **M**) Colony formation assays were used to evaluate the viability of SNU-387 and SNU-449. (**N**–**Q**) Wound healing and transwell assays were used to evaluate the migration ability of SNU-387 and SNU-449.

To further verify the biological role of LRRC59 in HCC, some experiments *in vitro* were performed. First, the mRNA protein of LRRC59 were detected in normal liver cell and HCC cell lines, the results indicated that its expression was upregulated in HCC cells compared to LO2 (Figure 8A–8C). Among these HCC cell lines, SNU-387 and SNU-449 exhibited the highest LRRC59 expression. Therefore, they were selected for subsequent experiments. Then LRRC59 was knockdown and verified by both qPCR and western blot in SNU-387 and SNU-449 (Figure 8D–8I). Functional assays including CCK-8 and colony formation assays revealed that LRRC59 knockdown undermined cell viability (Figure 8J–8M), the scratch assays and transwell assays demonstrated that LRRC59 knockdown impaired cell migration ability (Figure 8N–8Q).

### LRRC59 knockdown induces cell cycle arrest and apoptosis in HCC cells

**Figure 9:**
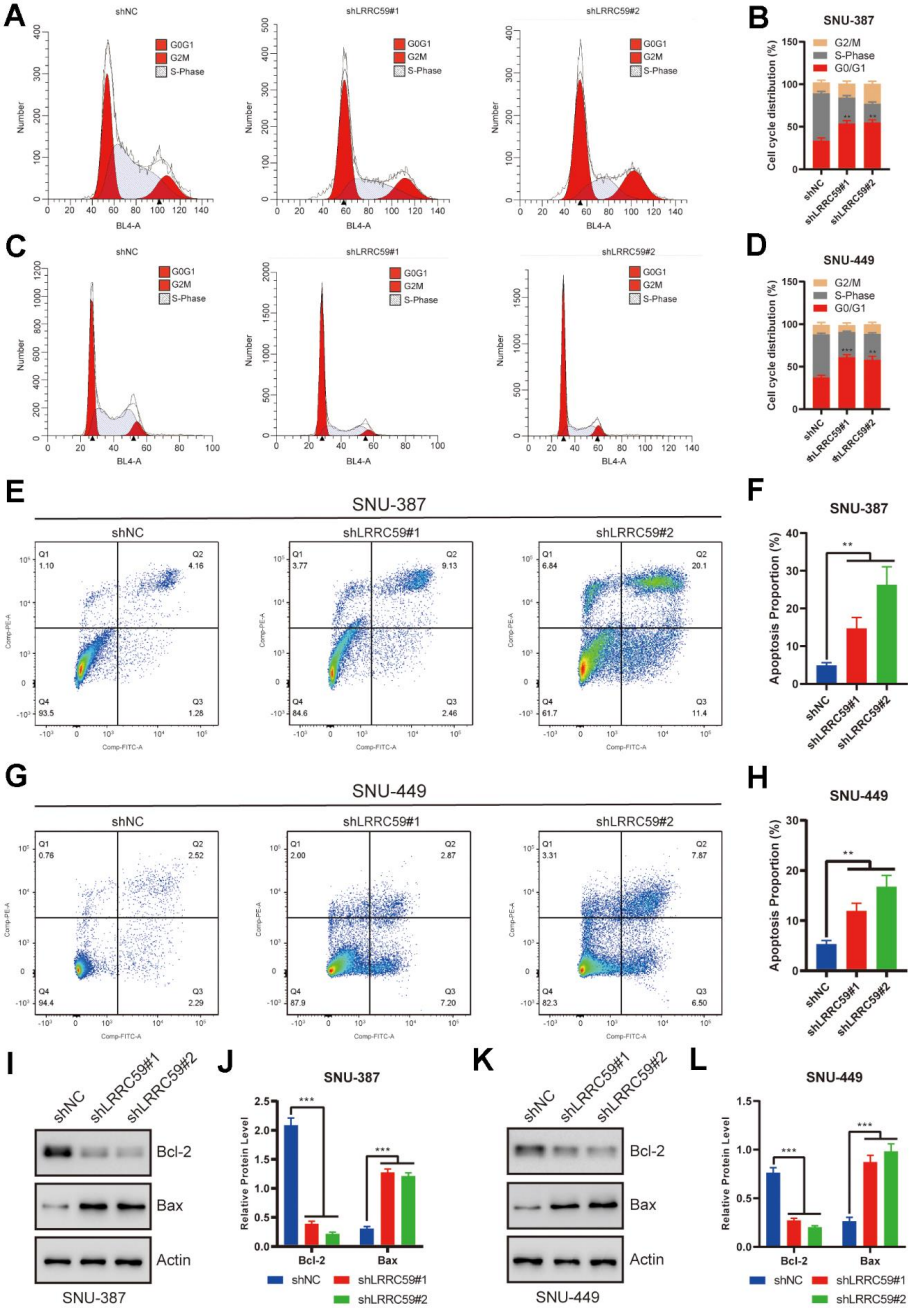
**LRRC59 knockdown induced cell cycle arrest and apoptosis in HCC cells.** (**A**–**D**) Flow cytometry was used to detect the role of LRRC59 on cell cycle arrest. (**E**–**H**) Flow cytometry was used to detect the role of LRRC59 on cell apoptosis. (**I**–**L**) Western blot showed the expression of apoptosis-related markers in SNU-387 and SNU-449.

The previous bioinformatics analysis showed a positive correlation between LRRC59 and cell cycle regulation. Therefore, we used flow cytometry to analyze the role of LRRC59 on the cell cycle. The results showed that LRRC59 knockdown in SNU-387 and SNU-449 induced G0/G1 arrest (Figure 9A–9D). Moreover, LRRC59 knockdown also promoted apoptosis in the HCC cells (Figure 9E–9H). Western blot results also showed that downregulated LRRC59 resulted in an increase in the expression of the pro-apoptotic protein Bax and a decrease in the expression of the anti-apoptotic protein Bcl-2 (Figure 9I–9L).

### LRRC59 knockdown enhances immunotherapy sensitivity in HCC cells

**Figure 10:**
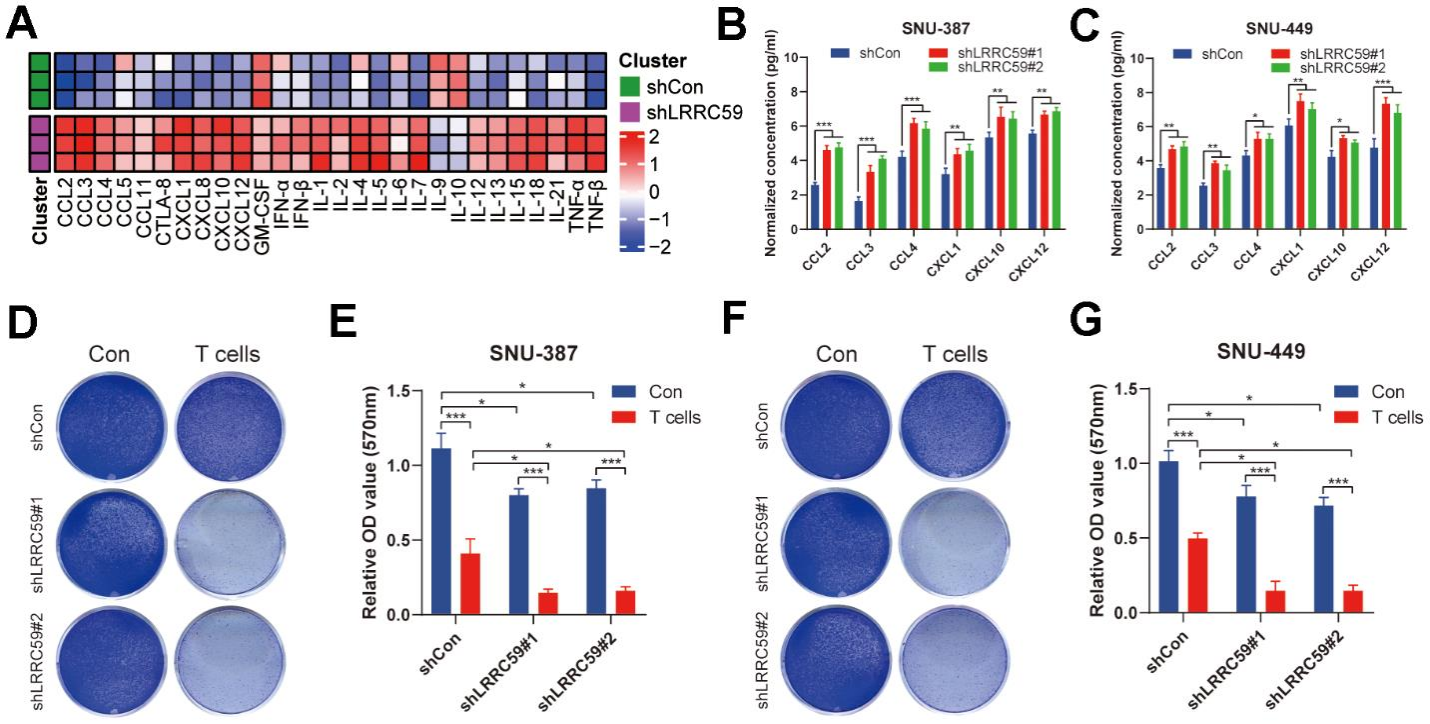
**LRRC59 knockdown rendered HCC cells more sensitive to immunotherapy.** (**A**) Heatmap of various cytokines and chemokines detected by ProcartaPlex multiple immunoassays between LRRC59 knockdown and the negative control groups in SNU387 cell culture supernatants. (**B**, **C**) The levels of some chemokines detected using ELISA between LRRC59 knockdown and the negative control groups in SNU-387 and SNU-449. (**D**–**G**) T cell-mediated tumor cell-killing assay between LRRC59 knockdown and negative control groups in SNU-387 and SNU-449.

Based on the previous immunoinformatics analysis, patients with high LRRC59 expression may have a poorer response to immunotherapy (Figure 7). We further validated it using in the HCC cells. First, ProcartaPlex multiple immunoassays between LRRC59 knockdown and the negative control groups in SNU-387 was performed to detect the chemokines and cytokines, and it showed that CCL2, CCL3, CCL4, CXCL1, CXCL10 and CXCL12 were significantly increased when LRRC59 was knockdown (Figure 10A). Moreover, the results of ELISA indicated that the protein level of these chemokines were obviously higher as well (Figure 10B, 10C). Finally, T cell-mediated tumor cell-killing assay was performed to detected the role of LRRC59 in attenuating the efficacy of immunotherapy, the results showed that LRRC59 knockdown enhances the efficacy of immunotherapy strongly (Figure 10D–10G). Taken together, these results suggested that targeting LRRC59 may benefit patients from immunotherapy.

## DISCUSSION

LRRC59 is a tail-anchored protein located to the endoplasmic reticulum and the nuclear envelope, with a transmembrane domain near its C-terminus [27]. It targets the ER membrane after translation and directly interacts with mRNA translation factors [28]. LRRC59 has recently been discovered to play an important role in regulating mRNA translation of secreted or membrane-encoded proteins through the SRP pathway [27, 28]. LRRC59 promotes cancer progression by regulating the nuclear transport of exogenous FGF1, and accelerates cancer cells proliferation and metastasis in various cancers [9, 29]. LRRC59 does not affect the translocation of FGF1 to the cytoplasm, but rather facilitates the import of cytoplasmic FGF1 to the nucleus through interactions with Kpns, coupled with LRRC59 movement along the ER and nuclear envelope membranes [9]. Moreover, LRRC59 in the ER may also be involved in protein ubiquitination, though the specific mechanism remains to be elucidated [7]. LRRC59 appears to be a potential biomarker in certain cancers, however, no study has performed an in-depth pan-cancer analysis of LRRC59 until now. In this study, we conducted a comprehensive bioinformatics analysis of LRRC59 using multiple public databases, aiming to systematically determine its expression patterns, prognostic value, and potential functions in pan-cancer.

Some studies have revealed that LRRC59 was overexpressed in several cancers and promoted tumor progression [7, 11, 12]. Consistent with these studies, our findings demonstrated significant overexpression of LRRC59 in most cancers compared to the corresponding normal tissues. Moreover, the expression levels of LRRC59 showed a positive correlation with clinical pathological stages in ACC, BLCA, ESCA, KICH, LIHC, LUAD, PAAD, THCA and UCS. And our prognosis analysis also revealed a negative correlation between LRRC59 expression levels and the prognosis of cancer patients in various types of cancer. Furthermore, our drug sensitivity analysis suggested that LRRC59 may reduce the sensitivity of cancer cells to chemotherapy drugs. Therefore, LRRC59 holds promise as a novel biomarker for pan-cancer diagnosis and prognosis.

The tumor immune microenvironment (TIME) constitutes a crucial component of the tumor microenvironment (TME) and is closely associated with tumor occurrence, development, metastasis, and drug resistance [30]. Tumor immunotherapy has advanced significantly in recent years. However, treatment efficacy still needs to be improved, necessitating the urgent identification of new therapeutic targets [31]. Our analysis of LRRC59 in relation to pan-cancer immune infiltration and immunotherapy reveals a positive correlation between LRRC59 expression and TIM-3 and TIGIT, while a negative correlation with LAG-3 and PDCD1. Immunotherapy targeting TIM-3 and TIGIT may be able to overcome immunotherapy resistance caused by LRRC59 overexpression.

Researches have indicated that LRRC59’s involvement in ER stress. The unfolded protein response (UPR) triggered by ER stress stands as a pivotal signaling pathway overseeing cellular adaptation to unfavorable microenvironments [32]. This pathway assumes a vital role in various cellular processes, such as tumor cell survival, and the maintenance of protein homeostasis [33]. Our enrichment analysis results revealed that LRRC59 was an important gene involved in protein synthesis and degradation within cancer cells. Because the liver is the most essential organ for protein synthesis in the body, amino acids absorbed from the digestive tract undergo processes like protein synthesis, deamination, and transamination in the liver, then the synthesized proteins enter the bloodstream to meet the demands of organs and tissues throughout the body, so it hints LRRC59 may play a critical role in the liver. Therefore, we chose HCC cells as the study object to further verify the biological function of LRRC59. Firstly, we used bioinformatics methods to find a positive correlation between LRRC59 expression and dendritic resting cell, as well as T regulatory cell in HCC, which meant that HCC patients with LRRC59 overexpression may be poor responsive to immunotherapy [34]. On the other hand, our *in vitro* experimental results confirmed that LRRC59 was overexpressed in HCC tissues and cells, which played a crucial role in promoting HCC cell proliferation, migration, and resistance to immunotherapy. Targeting LRRC59 may inhibit HCC progression and improve the efficacy of immunotherapy.

Although our study illustrates the multiple roles of LRRC59 in pan-cancer, it does have some limitations. Firstly, it remains uncertain how LRRC59 affects the efficacy of immunotherapy by modulating immune processes, although our results indicate that LRRC59 leads to immunotherapy resistance. Secondly, while our study may serve as a reference for future research, we did not conduct in-depth experiments to delve into the biological functional processes and molecular mechanisms about LRRC59 in pan-cancer.

Overall, we revealed that LRRC59 exhibits abnormal expression in cancer tissues and holds high diagnostic and prognostic value in this study. Additionally, we elucidated the role of LRRC59 in human cancer progression and treatment resistance through drug sensitivity analysis, enrichment analysis, mutation analysis, and immune analysis. We further validated these findings with a series of *in vitro* experiments in HCC. In summary, our research suggests that LRRC59 may serve as a potential novel prognostic marker and therapeutic target in pan-cancer.

## Supplementary Material

Supplementary Figures

Supplementary Table 1
