# Supplementary material for: Pan-cancer analysis of LRRC59 with a focus on prognostic and immunological roles in hepatocellular carcinoma
Source: Aging (Albany NY). 2024 May 10;16(9):8171–97. doi: 10.18632/aging.205810 (PMC11131990; doi:10.18632/aging.205810)
Supplement: Supplementary Figures [file aging-16-205810-s001.pdf]

SUPPLEMENTARY FIGURES

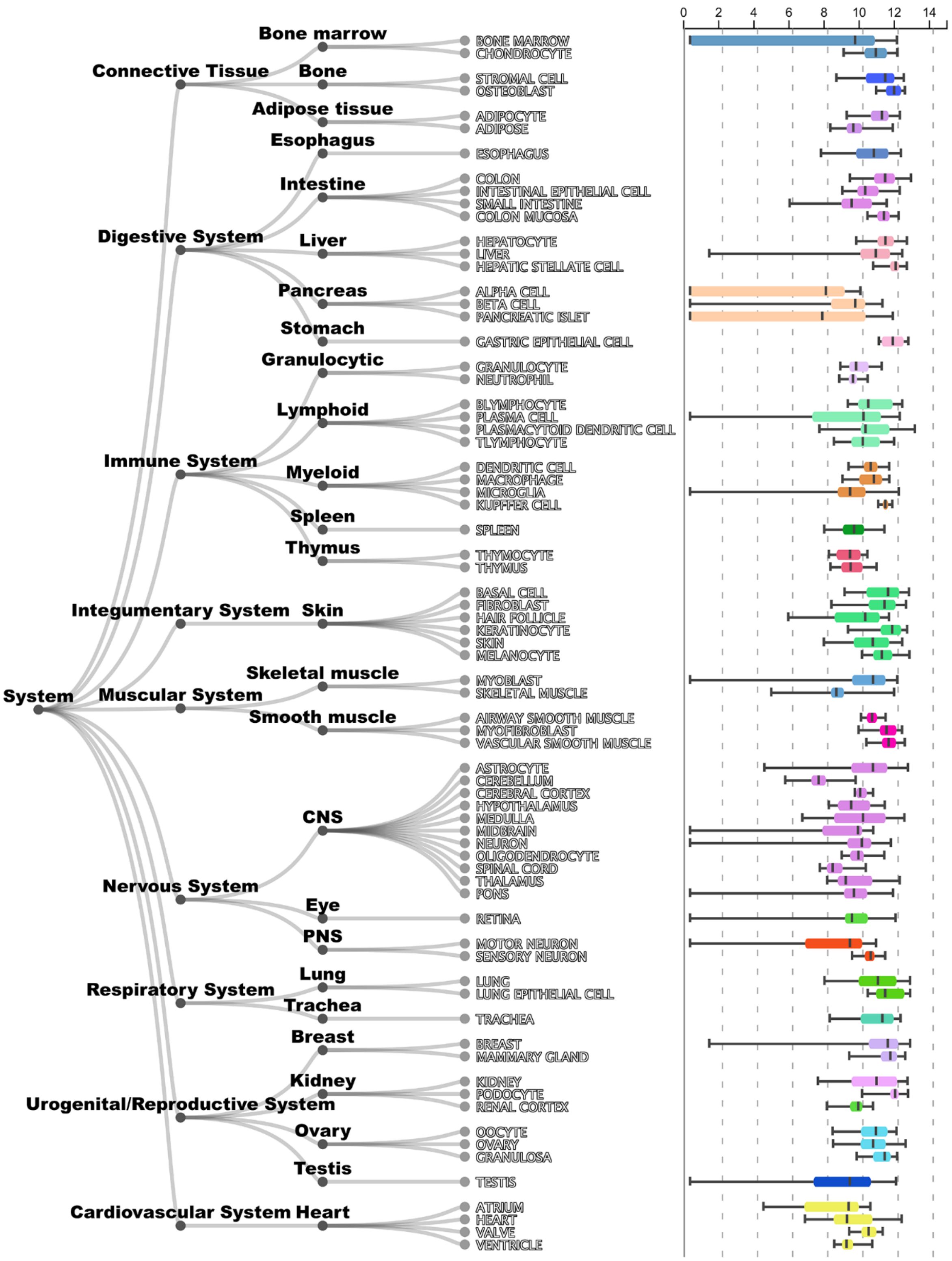

Supplementary Figure 1. The expression of LRRC59 in human normal tissues.

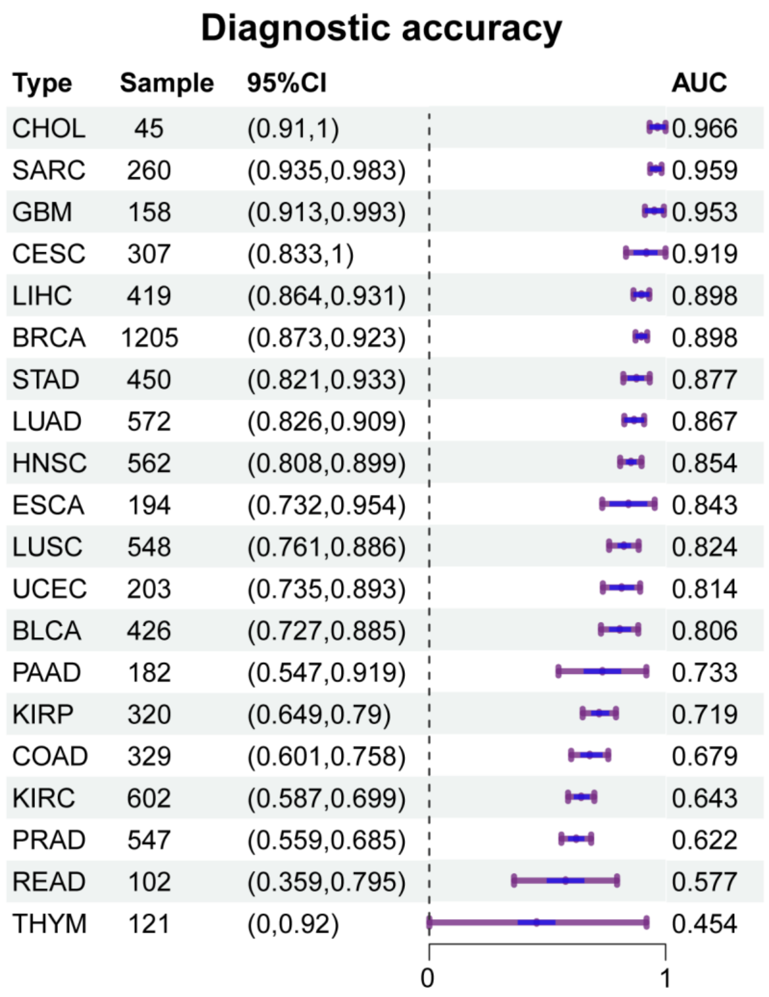

Supplementary Figure 2. The diagnostic accuracy of LRRC59 in various human tumors.

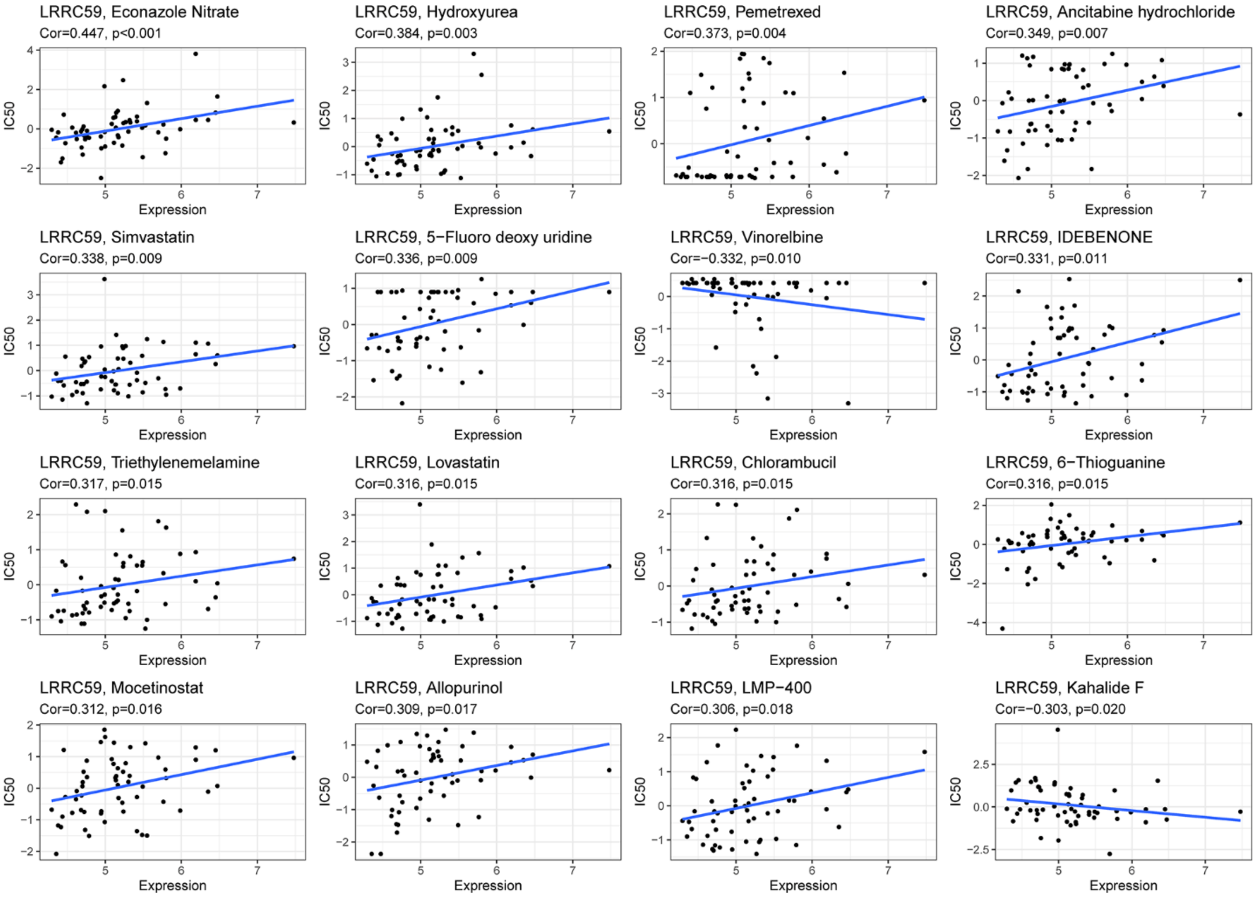

Supplementary Figure 3. Correlation analysis between LRRC59 expression and drug sensitivity.

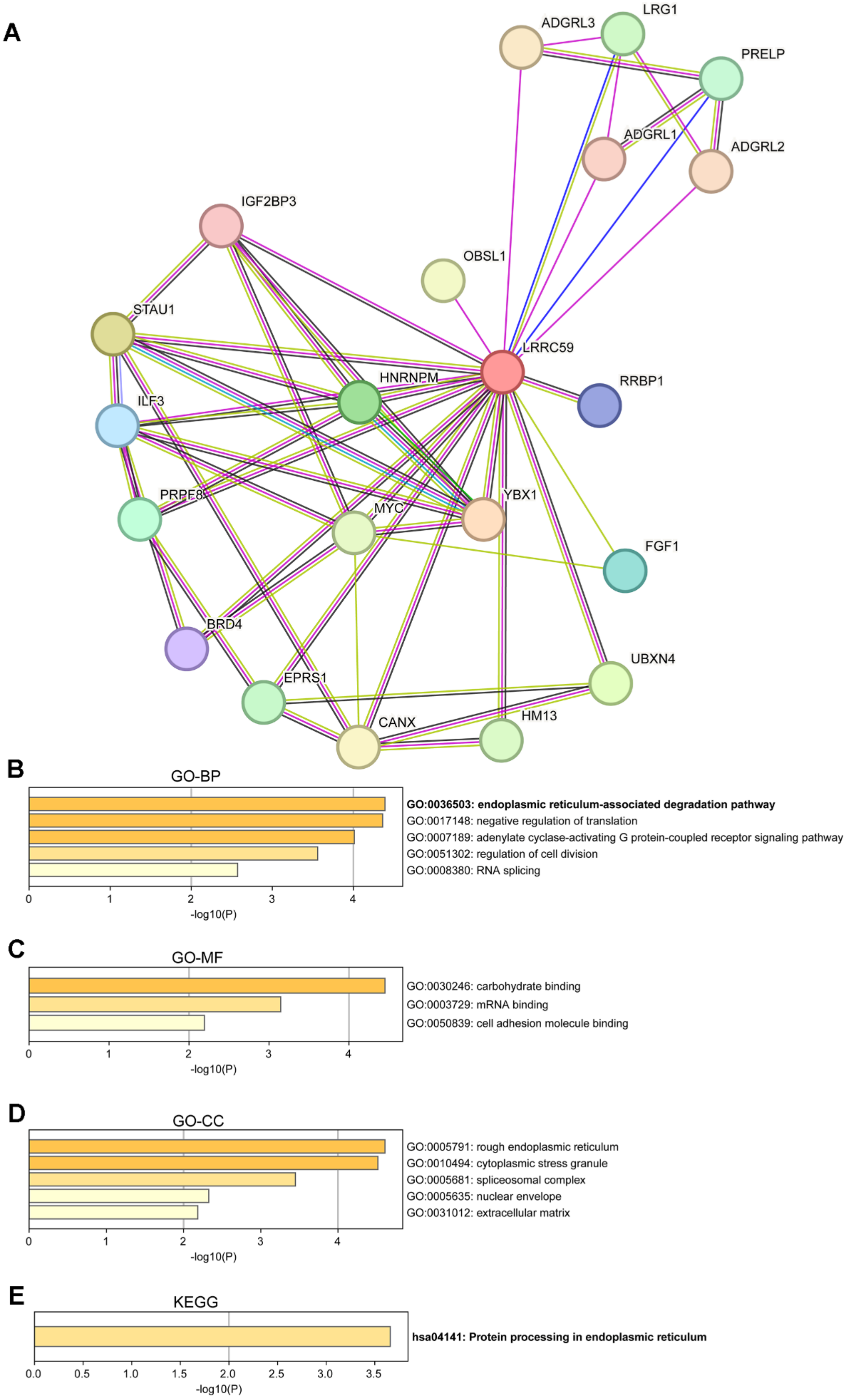

Supplementary Figure 4. The enrichment analysis of LRRC59 and its interaction genes. (**A**) LRRC59 and its 20 interacting genes from the STRING database. (**B**–**E**) The enrichment analysis of LRRC59 and its interaction genes.

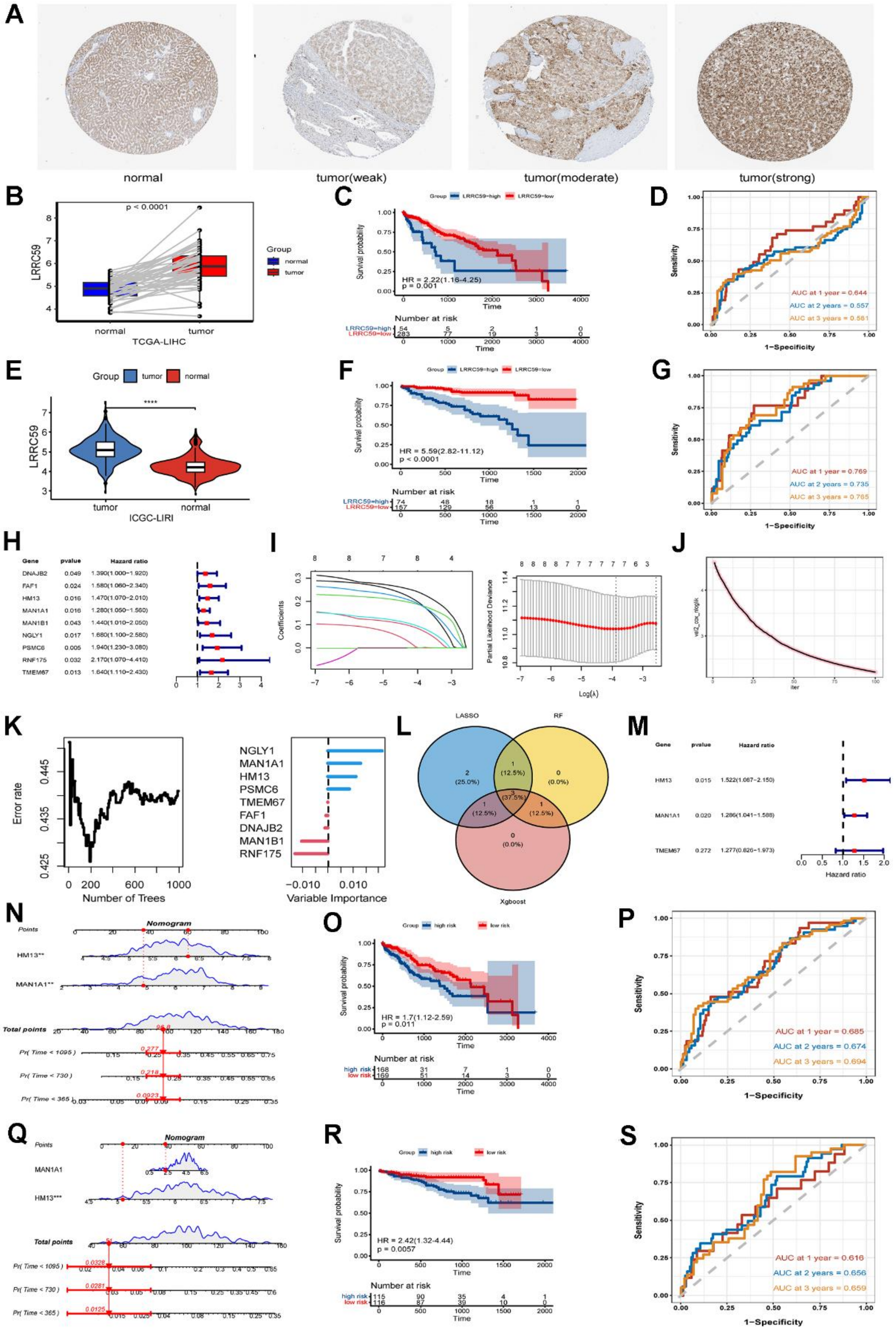

Supplementary Figure 5. The expression and prognosis of LRRC59 in HCC. (**A**) Immunohistochemical results of LRRC59 in liver normal tissues and HCC tissues. (**B**–**D**) LRRC59 paired expression analysis, prognosis analysis, and survival prediction analysis in TCGA-LIHC dataset. (**E**–**G**) LRRC59 expression analysis, prognosis analysis, and survival prediction analysis in ICGC-LIRI dataset. (**H**–**M**) Univariate Cox analysis, three machine learning algorithms, and multivariate Cox analysis were used in the TCGA-LIHC dataset to screen genes for subsequent modeled. (**N**–**P**) A predictive model was built in the TCGA-LIHC dataset and tested for the prognostic accuracy. (**Q**–**S**) A predictive model was built in the ICGC-LIRI dataset and tested for the prognostic accuracy.

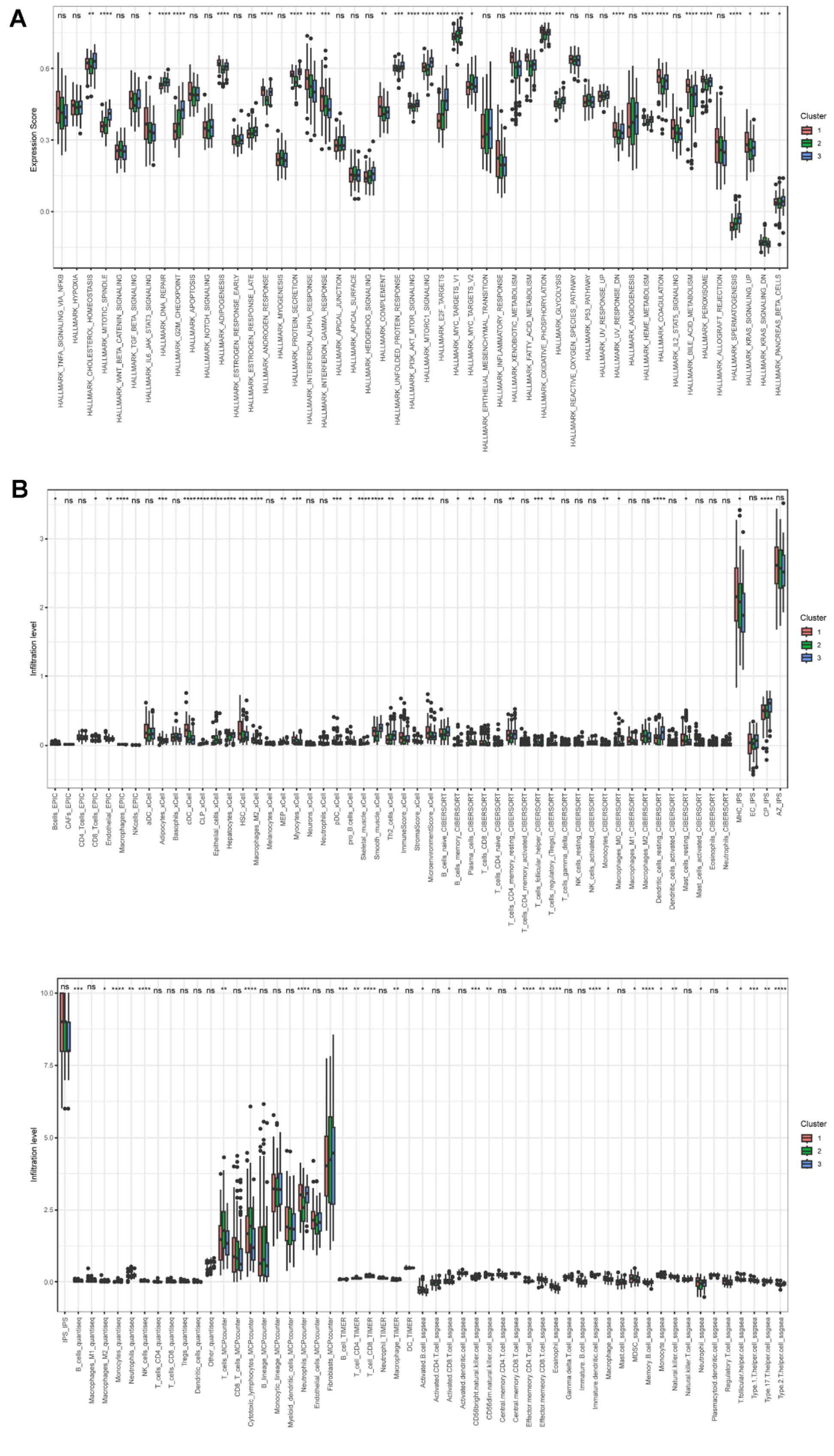

Supplementary Figure 6. Hallmark genomic score and immune cell infiltration differences among the three HCC subtypes. (**A**) Hallmark genomic score differences among the three HCC subtypes. (**B**) Differences in immune cell infiltration among the three HCC subtypes.

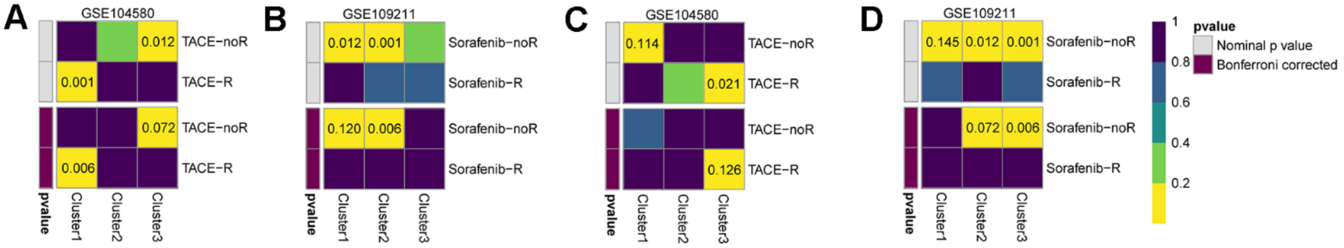

Supplementary Figure 7. The sensitivity to TACE and Sorafenib treatment of HCC subtypes. (**A**, **B**) Prediction of HCC subtypes and sensitivity to TACE and Sorafenib treatment in the ICGC-LIRI dataset. (**C**, **D**) Prediction of HCC subtypes and sensitivity to TACE and Sorafenib treatment in the TCGA-LIHC dataset.
