# Supplementary material for: Pan-cancer analysis of LRRC59 with a focus on prognostic and immunological roles in hepatocellular carcinoma
Source: Aging (Albany NY). 2024 May 10;16(9):8171–97. doi: 10.18632/aging.205810 (PMC11131990; doi:10.18632/aging.205810)
Supplement: Supplementary Table 1 [file aging-16-205810-s002.docx]

Supplementary Table 1. The genes of GOBP_ERAD_PATHWAY.

| TERM | GENE |
| --- | --- |
| GOBP_ERAD_PATHWAY | AMFR |
| GOBP_ERAD_PATHWAY | ANKZF1 |
| GOBP_ERAD_PATHWAY | AQP11 |
| GOBP_ERAD_PATHWAY | ATF6 |
| GOBP_ERAD_PATHWAY | ATXN3 |
| GOBP_ERAD_PATHWAY | AUP1 |
| GOBP_ERAD_PATHWAY | BAG6 |
| GOBP_ERAD_PATHWAY | BCAP31 |
| GOBP_ERAD_PATHWAY | BRSK2 |
| GOBP_ERAD_PATHWAY | CALR |
| GOBP_ERAD_PATHWAY | CALR3 |
| GOBP_ERAD_PATHWAY | CANX |
| GOBP_ERAD_PATHWAY | CAV1 |
| GOBP_ERAD_PATHWAY | CCDC47 |
| GOBP_ERAD_PATHWAY | CLGN |
| GOBP_ERAD_PATHWAY | DERL1 |
| GOBP_ERAD_PATHWAY | DERL2 |
| GOBP_ERAD_PATHWAY | DERL3 |
| GOBP_ERAD_PATHWAY | DNAJB12 |
| GOBP_ERAD_PATHWAY | DNAJB2 |
| GOBP_ERAD_PATHWAY | DNAJB9 |
| GOBP_ERAD_PATHWAY | DNAJC10 |
| GOBP_ERAD_PATHWAY | ECPAS |
| GOBP_ERAD_PATHWAY | EDEM1 |
| GOBP_ERAD_PATHWAY | EDEM2 |
| GOBP_ERAD_PATHWAY | EDEM3 |
| GOBP_ERAD_PATHWAY | ERLEC1 |
| GOBP_ERAD_PATHWAY | ERLIN1 |
| GOBP_ERAD_PATHWAY | ERLIN2 |
| GOBP_ERAD_PATHWAY | FAF1 |
| GOBP_ERAD_PATHWAY | FAF2 |
| GOBP_ERAD_PATHWAY | FAM8A1 |
| GOBP_ERAD_PATHWAY | FBXO17 |
| GOBP_ERAD_PATHWAY | FBXO2 |
| GOBP_ERAD_PATHWAY | FBXO27 |
| GOBP_ERAD_PATHWAY | FBXO44 |
| GOBP_ERAD_PATHWAY | FBXO6 |
| GOBP_ERAD_PATHWAY | FOXRED2 |
| GOBP_ERAD_PATHWAY | GET4 |
| GOBP_ERAD_PATHWAY | HERPUD1 |
| GOBP_ERAD_PATHWAY | HM13 |
| GOBP_ERAD_PATHWAY | HSP90B1 |
| GOBP_ERAD_PATHWAY | HSPA5 |
| GOBP_ERAD_PATHWAY | JKAMP |
| GOBP_ERAD_PATHWAY | MAN1A1 |
| GOBP_ERAD_PATHWAY | MAN1B1 |
| GOBP_ERAD_PATHWAY | MARCHF6 |
| GOBP_ERAD_PATHWAY | NCCRP1 |
| GOBP_ERAD_PATHWAY | NFE2L2 |
| GOBP_ERAD_PATHWAY | NGLY1 |
| GOBP_ERAD_PATHWAY | NPLOC4 |
| GOBP_ERAD_PATHWAY | OS9 |
| GOBP_ERAD_PATHWAY | PRKN |
| GOBP_ERAD_PATHWAY | PSMC6 |
| GOBP_ERAD_PATHWAY | RCN3 |
| GOBP_ERAD_PATHWAY | RHBDD1 |
| GOBP_ERAD_PATHWAY | RHBDD2 |
| GOBP_ERAD_PATHWAY | RNF103 |
| GOBP_ERAD_PATHWAY | RNF121 |
| GOBP_ERAD_PATHWAY | RNF139 |
| GOBP_ERAD_PATHWAY | RNF175 |
| GOBP_ERAD_PATHWAY | RNF185 |
| GOBP_ERAD_PATHWAY | RNF5 |
| GOBP_ERAD_PATHWAY | RNFT1 |
| GOBP_ERAD_PATHWAY | RNFT2 |
| GOBP_ERAD_PATHWAY | SDF2L1 |
| GOBP_ERAD_PATHWAY | SEC61B |
| GOBP_ERAD_PATHWAY | SEL1L |
| GOBP_ERAD_PATHWAY | SEL1L2 |
| GOBP_ERAD_PATHWAY | SELENOS |
| GOBP_ERAD_PATHWAY | SGTA |
| GOBP_ERAD_PATHWAY | STT3B |
| GOBP_ERAD_PATHWAY | STUB1 |
| GOBP_ERAD_PATHWAY | SVIP |
| GOBP_ERAD_PATHWAY | SYVN1 |
| GOBP_ERAD_PATHWAY | TMEM129 |
| GOBP_ERAD_PATHWAY | TMEM259 |
| GOBP_ERAD_PATHWAY | TMEM67 |
| GOBP_ERAD_PATHWAY | TMUB1 |
| GOBP_ERAD_PATHWAY | TMUB2 |
| GOBP_ERAD_PATHWAY | TOR1A |
| GOBP_ERAD_PATHWAY | TRIM13 |
| GOBP_ERAD_PATHWAY | TRIM25 |
| GOBP_ERAD_PATHWAY | UBAC2 |
| GOBP_ERAD_PATHWAY | UBE2G2 |
| GOBP_ERAD_PATHWAY | UBE2J1 |
| GOBP_ERAD_PATHWAY | UBE2J2 |
| GOBP_ERAD_PATHWAY | UBE4A |
| GOBP_ERAD_PATHWAY | UBE4B |
| GOBP_ERAD_PATHWAY | UBQLN1 |
| GOBP_ERAD_PATHWAY | UBQLN2 |
| GOBP_ERAD_PATHWAY | UBXN1 |
| GOBP_ERAD_PATHWAY | UBXN10 |
| GOBP_ERAD_PATHWAY | UBXN4 |
| GOBP_ERAD_PATHWAY | UBXN6 |
| GOBP_ERAD_PATHWAY | UBXN8 |
| GOBP_ERAD_PATHWAY | UFD1 |
| GOBP_ERAD_PATHWAY | UGGT1 |
| GOBP_ERAD_PATHWAY | UGGT2 |
| GOBP_ERAD_PATHWAY | UMOD |
| GOBP_ERAD_PATHWAY | USP13 |
| GOBP_ERAD_PATHWAY | USP14 |
| GOBP_ERAD_PATHWAY | USP19 |
| GOBP_ERAD_PATHWAY | USP25 |
| GOBP_ERAD_PATHWAY | VCP |
| GOBP_ERAD_PATHWAY | WFS1 |
| GOBP_ERAD_PATHWAY | XBP1 |
| GOBP_ERAD_PATHWAY | YOD1 |
